# The Management of Abortions

**Published:** 1892-04

**Authors:** J. W. Carhart

**Affiliations:** Lampasas, Texas


### DANIEL'S
### TEXAS MEDICAL JOURNAL.

A Representative Organ of the Medical Profession, and an Exponent of Rational Medicine; devoted to the Organization, Advancement and Elevation of the Profession in Texas.

PUBLISHED MONTHLY.—SUBSCRIPTION $2.00 A YEAR.

Vol. VII. AUSTIN, APRIL, 1892. No. 10.

### Original Articles.

CONTRIBUTED EXCLUSIVELY TO THIS JOURNAL.

*The Articles in this Department are accepted and published with the understanding that we are not responsible for, nor do we indorse the views and opinions of the writers by so doing.*

For Daniel's Texas Medical Journal.

### THE MANAGEMENT OF ABORTIONS.

BY J. W. CARHART, M. D., LAMPASAS, TEXAS.

[Read at Meeting Austin District Medical Association.]

THIS is one of those medical subjects upon which there has been considerable talk, and but little said. I am not sure that my talk will add much, if anything, to the accumulated wisdom on this subject.

We will at least be entitled to the merit of brevity, if not for wisdom and originality.

The opinions of practitioners on the subject of "the management of abortions," are almost as numerous and varied as those who hold them. The teachings of authors are not by any means uniform touching this subject; and these facts go to show that circumstances differ in cases of abortion, as in most other matters of medical practice and surgery; and it is therefore impossible to lay down any hard and fast rules for the guidance of practitioners, and, in a certain sense, and to a considerable extent, every man must, of necessity, be "a law unto himself." In other words, after one has carefully studied authorities on the subject, he must make careful observations and exercise good common sense. If he is incapable of this, as, alas! some are, he should practice farming instead of obstetrics.

To come, then, to the subject in hand, it would be competent for us to say, in the first place, that there are two kinds of abortions: the intentional and the accidental.

Since intentional abortions constitute a class by themselves, it is fair to presume that the committee, in awarding us this subject, expected us to deal with the accidental class, except, perhaps, as it regards detail in after particulars, where, of course, the management would be practically the same.

The first proposition that we would enunciate in regard to the accidental class is, try and prevent them.

If called too late, preventive measures would, of course, not be justifiable. If called in time, and the case is properly diagnosed, sulphate of morphia may be injected hypodermically, and the pains stopped. Frequently nothing more will be needed, except to order the patient to bed, or in the recumbent position, if not already there, and enjoin perfect quiet. If there be any specially exciting cause, so far as possible, see that it is removed. If there be mental disturbance, give five drops of tincture hyoscyamus in water, or the bromides. If, on examination, the womb appears lax and the os distensible, give ergot and tincture of black haw. These remedies I have found of more service in these cases than any and all else.

If the abortion has actually taken place when we reach the patient, or the process cannot be stopped, and we find that it is bound to come, why, let it come, and take good care of the woman.

First, by keeping her absolutely quiet until the danger of flooding is past. Guard her carefully against peritonitis by preventing her taking cold and by keeping the lower bowel free from obstruction. This can generally be done by enemas of very warm water. Do not be afraid of warm water for this purpose. It will do good in several particulars.

Give vaginal injections of warm water and some mild, chemican antiseptic, as often as is thought necessary. Do not allow the conjugal approaches of the husband for some time after the occurrence, and not until you are confident that the normal condition of the patient is established. I need not detail all the reasons for this.

I am satisfied that the point the committee chiefly wanted discussed was the question of the disposal of the uterine secundines after abortion.

Of course, the fœtus generally comes away as the result of uterine contraction. Some times it has to be removed by surgical interference. In that case, do the best you can. What would do in one case, would not be allowable in another. Here knowledge and common sense are worth more than all else. Never be in too big a hurry. Do not condemn a professional brother who may not see fit to do just as you would do--with a case of your own not exactly like his.

The question arises, what would you do about the uterine secundines?

I answer, I would do the best I could, under all the circumstances. If they came away in good time of their own accord, I would consider myself, and particularly the woman, very fortunate, and would congratulate her on her good fortune, and not myself on my skill in delivering secundines.

But suppose you have a case where the fœtus had already been discharged, and an hour or two had elapsed, and the womb had contracted down quite solidly, the os closing hard about the cord —the woman having lost considerable blood, and somewhat exhausted from pain, the afterbirth grown fast, as it generally is in the first three months of pregnancy,—what would you do about it?

The answers from you, gentlemen, would be somewhat varied. One doctor is reported to have cut out an afterbirth, in such a case, with a knife. Another invariably gives chloroform to anæsthesia,—dilates the womb and removes the secundines, with finger if he can, with some sort of an instrument if he must. The afterbirth must come away, right away.

I would do no such thing. I wold let it alone, and allow nature to expel it, and watch my opportunity to assist nature and help the woman all I could without doing any harm.

Many a woman has been sacrificed by too much rashness in removing secundines after abortion. To sustain my position in this matter, I call your attention to several authorities.

Barton Cook Hurst, M. D., of Philadelphia, says, in "System of Obsteterics by American Authors," Vol. 1, p. 311, "Most frequently it is the embryo alone, or at most the ovum, in whole or in part, covered often by the ovular decidua that is cast off, while the uterine decidua remain behind within the uterus."

Duhressen, from a rich experience in the service of the Chariti in Berlin, says, "The retention of the portions of the secundines —the decidua vera is not the exception but the rule." And Tarnier says that "ordinarily the uterine decidua remain adherent to the uterus."

I am aware of the danger of septicæmia in cases of retained afterbirth, but that, fortunately, does not often occur.

If an effort were made to deliver the placenta with instruments, with the woman under chloroform, there would be no means of avoiding injury to the endometrium, which would be the source of greater danger than to allow the afterbirth to remain.

I will next quote from one of the most thoroughly scientific writers on this subject, viz: William Leishman, M. D., as found in his work entitled "A System of Midwifery," p. 366: "In all cases the placenta is retained much longer after the expulsion of the child in abortion than in labor at full term."

Thus wrote Burns, and his assertion is undoubtedly correct: . . . "The uterine contractions suffice, in many instances, to burst the ovum and discharge the fœtus, and when the cord breaks or is tied, uterine action ceases. But instead of a speedy recurrence of the pains and a natural and unaided expulsion of the placenta with the membranes, it is retained, sometimes for hours only, but oftener for a much longer period, extending to eight or ten days, or even more. . . . . A return of the pains after a very variable interval, marks a renewed attempt on the part of the uterus to rid itself of its contents. If a considerable time should elapse the os will have closed so firmly that a tedious process, which is conducted at great mechanical disadvantage, is necessary for its dilatation. . . . . The structures of the afterbirth may be broken up and discharged piecemeal; but the process is always tedious and may be accompanied by low fever . . . . and there is of course the danger of what fortunately does not often occur in such cases, viz: blood poisoning through the uterine veins."

At the risk of wearying you, I quote one more eminent authority to show that the greatest care should be exercised in the use of instruments in the removal of retained placenta after abortion.

Henry D. Fry, M. D., in the "American Journal of Obstetrics," Vol. 2, p. 593, advocates the use of electricity as a substitute for the curette (about the only other instrument that ever should be used) in incomplete abortion. He remarks as to the curette that "its action cannot be limited to the tissues we wish to remove, but it will scrape healthy tissue also. For the immediate removal of retained secundines the faradic current is employed, but for the removal of these after retention for some time, the galvanic current should be used. He reports a case in which the latter current was used successfully, the strength being sixty milliamperes continued for eight minutes and repeated three times. The positive pole was introduced into the uterus, the selection being made because of the local effect, because this pole promotes coagulation, and because it is hæmostatic; a fourth reason is added as probable but not proven—its antiseptic powers."
—Annual of the Universal Medical Sciences, Vol. II, pp. 2, 3.

I could give many more authorities, but if the foregoing are not sufficient, more would do no good.

